# In Situ Synthesis of RMB_6_-TMB_2_ Composite Nanopowders via One-Step Solid-State Reduction

**DOI:** 10.3390/nano15171341

**Published:** 2025-09-01

**Authors:** Xiaogang Guo, Linyan Wang, Hang Zhou, Jun Xu, An Liu, Mengdong Ma, Rongxin Sun, Weidong Qin, Yufei Gao, Bing Liu, Baozhong Li, Lei Sun, Dongli Yu

**Affiliations:** 1 Center for High Pressure Science (CHiPS), State Key Laboratory of Metastable Materials Science and Technology, Yanshan University, Qinhuangdao 066004, China; guoxiaogang1994@stumail.ysu.edu.cn (X.G.); zh18993920050@stumail.ysu.edu.cn (H.Z.); xujun0924@stumail.ysu.edu.cn (J.X.); weidong07080814@stumail.edu.cn (W.Q.); gyf@ysu.edu.cn (Y.G.); lbz@ysu.edu.cn (B.L.); ydl@ysu.edu.cn (D.Y.); 2 State Key Laboratory of Advanced Space Propulsion, Space Engineering University, Beijing 101416, China; wanglinyan@hgd.edu.cn; 3 State Key Laboratory of Crane Technology, Yanshan University, Qinhuangdao 066004, China; liuan@stumail.ysu.edu.cn (A.L.); mamengdong@ysu.edu.cn (M.M.); 4 Super Hard Material Industry Technology Research Institute, Zhengzhou 450000, China; sunrongxin@hnas.ac.cn

**Keywords:** RMB_6_-TMB_2_, composite nanopowder, in situ synthesize, homogeneous phase distribution

## Abstract

RMB_6_-TMB_2_ (RM = rare earth elements, TM = transition metal elements) composites retain superior field emission properties of RMB_6_ while addressing its inherent mechanical limitations by constructing a eutectic structure with TMB_2_. Herein, an in situ route for synthesizing RMB_6_-TMB_2_ composite nanopowders with homogeneous phase distribution using reduction reactions was proposed. The LaB_6_-ZrB_2_ composite nanopowders were synthesized in situ for the first time using sodium borohydride (NaBH_4_) as both a reducing agent and boron source, with lanthanum oxide (La_2_O_3_) and zirconium dioxide (ZrO_2_) serving as metal sources. The effects of the synthesis temperature on phase compositions and microstructure of the composites were systematically investigated. The LaB_6_-ZrB_2_ system with a eutectic weight ratio exhibited an accelerated reaction rate, achieving a complete reaction at 1000 °C, 300 °C lower than that of single-phase ZrB_2_ synthesis. The composite phases were uniformly distributed even at nanoscale. The composite powder displayed an average particle size of ~170 nm when synthesized at 1300 °C. With the benefit of the in situ synthesis method, LaB_6_-TiB_2_, CeB_6_-ZrB_2_, and CeB_6_-TiB_2_ composite powders were successfully synthesized. This process effectively addresses phase separation and contamination issues typically associated with traditional mixing methods, providing a scalable precursor for high-performance RMB_6_-TMB_2_ composites.

## 1. Introduction

Metal boride materials have great potential for various applications due to their diverse crystalline structures, stoichiometric compositions, and excellent physical and chemical features [1,2,3,4,5,6,7,8]. Among these, rare-earth hexaborides (RMB_6_, where RM = La, Ce, Pr, Nd, etc.) have drawn significant attention due to their excellent electron emission properties. In particular, lanthanum hexaboride (LaB_6_) and cerium hexaboride (CeB_6_), with their relatively low work function of 2.1–2.8 eV and 2.2–2.8 eV, respectively, have become ideal materials for advanced cathode applications [9,10,11,12,13,14,15,16,17,18,19]. Until now, LaB_6_ and CeB_6_ single crystals have been widely employed as electron emission cathodes and other vacuum electron devices [20]. Nevertheless, their practical applications are often constrained by inherent brittleness and size limitations, which pose significant challenges for their manufacture and application [21,22,23].

It has been observed that polycrystalline materials, particularly nanocrystals, can overcome the inherent defects of single crystals, resulting in enhanced mechanical properties. For example, the fracture toughness of LaB_6_ polycrystals (3.0–3.2 MPa·m^1/2^) is 67–78% larger than single crystals (1.8 MPa·m^1/2^) [9,24,25,26,27]. Additionally, LaB_6_ nanocrystalline ceramics exhibit a thermal field emission performance that is comparable to single crystals, with an emission current density of 34.8 A/cm^2^, which is 92% of the single crystal value (37.8 A/cm^2^) [28]. In addition, adding a secondary phase can further enhance the mechanical properties of LaB_6_ ceramics on the basis of fine-grained strengthening. Recent investigations have shown that the integration of transition metal diboride (TMB_2_, where TM = Zr, Ti, etc.) into LaB_6_ substrates can significantly improve mechanical properties while maintaining their superior thermal emission properties. Notably, the formation of a eutectic structure with a weight ratio of 79:21 (RMB_6_:TMB_2_) has shown remarkable improvements in both strength and ductility [29,30,31,32,33]. For example, LaB_6_-ZrB_2_ composites with a eutectic ratio exhibit enhanced fracture toughness (up to ~3.7 MPa·m^1/2^) compared to LaB_6_ polycrystals [34]. However, micron-scale precursors and blending techniques (e.g., mechanical mixing or ultrasonic agitation) have difficulty achieving sufficient uniformity or may introduce impurities, which directly impairs the performance of the final composites. It has been demonstrated that the in situ synthesis method achieves a nearly homogeneous phase distribution, effectively eliminates impurity introduction, and simplifies experimental procedures [35,36]. To our knowledge, no studies have reported on the synthesis method for RMB_6_-TMB_2_ composite powders. However, both individual boride nanoparticles (RMB_6_ and TMB_2_) can be successfully synthesized using NaBH_4_ as the reducing agent [13,30,37,38,39,40,41].

In this work, we introduce a novel in situ approach to fabricate RMB_6_-TMB_2_ composite powders via a one-step solid-state reduction process using metal oxides and sodium borohydride. We systematically investigated how synthesis temperature affects both the phase composition and particle morphology of the LaB_6_-ZrB_2_ composite powders. It has been observed that the complete reaction temperature for the eutectic ratio mixture is lower than that of the single-phase TMB_2_. Furthermore, the introduction of TMB_2_ partially suppresses the growth rate of RMB_6_-TMB_2_ grains, with abnormal grain growth occurring at 1300 °C. Then, LaB_6_-TiB_2_, CeB_6_-ZrB_2_, and CeB_6_-TiB_2_ composite powders were successfully prepared using NaBH_4_, confirming the universality of this method in the preparation of RMB_6_-TMB_2_. The resulting powders have a homogeneous phase distribution and nanosized particles, making them highly suitable applications in electronics, energy storage devices, and other high-performance technologies.

## 2. Experiment

The metal oxides included La_2_O_3_ powder (99.99% purity, 1–3 μm; Qinhuangdao ENO High-Tech Material Development Co., Ltd., Qinhuangdao, China), CeO_2_ powder (99.9% purity, 3–5 μm; InnoChem Science & Technology Co., Ltd., Beijing, China), and ZrO_2_ powder (99.99% purity, 50 nm; Shanghai Macklin Biochemical Co., Ltd., Shanghai, China); TiO_2_ powder (99.99% purity, 50 nm; Shanghai Macklin Biochemical Co., Ltd., Shanghai, China) acted as the metal source, whereas NaBH_4_ powder (99.99% purity; InnoChem Science & Technology Co., Ltd., Beijing, China) served as both the reducing agent and boron source. Metal oxides and NaBH_4_ were manually stirred in an agate mortar for 30 min to ensure intimate contact between reactants. The raw materials of the composite nanopowders were mixed according to the eutectic weight ratio of 79:21 (RMB_6_:TMB_2_). To ensure complete conversion of the raw materials, the molar ratio of NaBH_4_ was doubled compared with its theoretical requirement (La_2_O_3_:NaBH_4_ = 1:26, CeO_2_:NaBH_4_ = 3:40, and ZrO_2_/TiO_2_:NaBH_4_ = 1:6). The homogeneous mixtures were uniaxially pressed into cylindrical pellets (Φ30 mm × 5 mm) under 5 MPa of pressure and then covered with graphite foil. The wrapped pellets were treated in a tube furnace under argon (99.99% purity, 10 mL/min). The reactants were heated from room temperature to 120 °C over a constant heating rate of 10 °C/min, followed by a three-hour dwell to remove hydroxides and moisture. Then, further heating was carried out to target temperatures (800–1300 °C) at the same rate, holding for 1 h to ensure a complete reaction. After cooling, the reacted product was manually ground and pulverized into powders using an agate mortar. To remove byproducts (e.g., NaBO_2_, Na), the powders were magnetically stirred in deionized water for 2 h, followed by centrifugation at 3000 rpm for 10 min. The above washing operation was repeated 3–5 times, and the final product was obtained after drying at 80 °C for 6 h in a vacuum oven.

The phases in the synthesized nanopowders were detected via an X-ray diffractometer (XRD; DMAX2500/P, Rigaku, Akishima, Japan) working at an accelerating voltage of 40 kV and an emission current of 30 mA at a scan rate of 4°/s and a step size of 0.01° from 20–60° with Cu Kα radiation (*λ* = 1.5406 Å). The Rietveld refinement was conducted using the FullProf suit software package (v.5, GSAS). The phase compositions and microstructure of the powders were characterized using a scanning electron microscope (SEM; Verios G4 UC, Thermo Fisher Scientific, Waltham, MA, USA) equipped with an energy dispersive spectroscopy system (EDS; Ultim Extreme, Oxford Instruments, Abingdon, Oxfordshire, UK) at an accelerating voltage of 2–15 kV, and a transmission electron microscope (TEM; Talos F200X, Thermo Fisher Scientific, Waltham, MA, USA) paired with energy dispersive spectroscopy (EDS).

## 3. Results and Discussion

**Figure 1:**
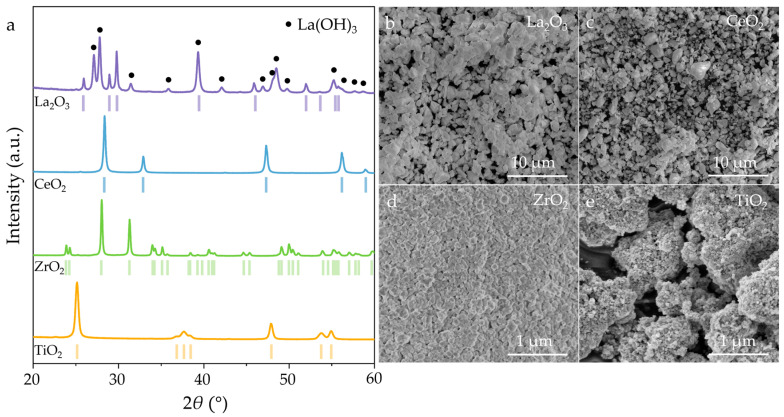
(**a**) XRD patterns and (**b**–**e**) SEM images of La_2_O_3_, CeO_2_, ZrO_2_, and TiO_2_ powders.

The SEM images and XRD patterns of the metal oxides are shown in Figure 1. The XRD patterns (Figure 1a) confirmed the absence of impurities in the CeO_2_ (PDF#97-016-7160), ZrO_2_ (PDF#97-009-6537), and TiO_2_ (Ti_0.72_O_2_, PDF#97-008-2084) powders, whereas La(OH)_3_ (PDF#97-024-5670) is detected in the La_2_O_3_ (PDF#97-005-6166) powder due to its hygroscopicity. Therefore, drying is necessary to remove hydroxides and the absorbed water from the powder prior to further processing. SEM images (Figure 1b–e) reveal that the particles of the RMB_6_ (La_2_O_3_ and CeO_2_) precursors have sizes within a range of several microns (1–5 μm), and the precursors of TMB_2_ (ZrO_2_ and TiO_2_) consist of agglomerated nanoparticles (~50 nm).

To synthesize composite powders, a single-phase system was employed to discuss the feasibility of this method. Single-phase LaB_6_ and ZrB_2_ nanopowders were synthesized via a reaction between their precursor (La_2_O_3_ or ZrO_2_) and NaBH_4_ from 800 °C to 1300 °C. The reaction between La_2_O_3_ and NaBH_4_ is depicted in Equation (1) [30,42]:

(1) $${La}_{2}O_{3}\;+\;13NaBH_{4}\to 2LaB_{6}\;+\;NaBO_{3}\;+\;12Na\;+\;26H_{2}\;$$

(2) $$NaBH_{4}\to BH_{3}\;+\;NaH$$

(3) $${La}_{2}O_{3}\;+\;13BH_{3}\;+\;13NaH\to 2LaB_{6}\;+\;NaBO_{3}\;+\;12Na\;+\;26H_{2}$$

**Figure 2:**
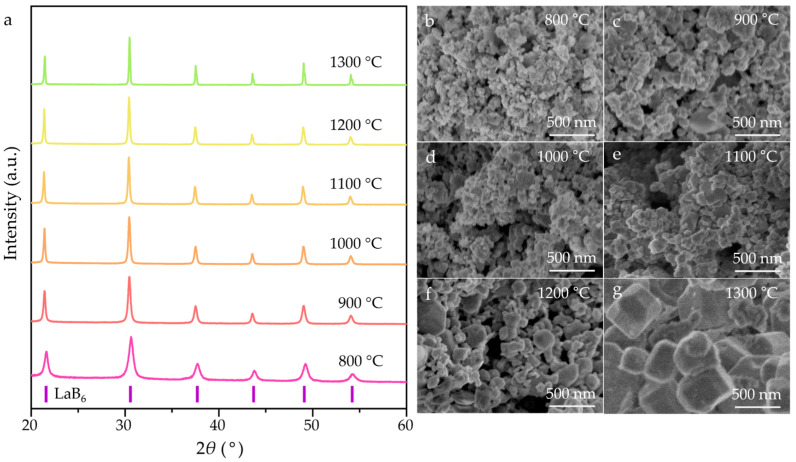
(**a**) XRD patterns and (**b**–**g**) SEM images of LaB_6_ nanopowders synthesized at 800–1300 °C.

Initially, NaBH_4_ decomposes to BH_3_ and NaH when reaching 500 °C, according to Equation (2) [43]. Then, LaB_6_ was synthesized via a reaction between BH_3_, NaH, and La_2_O_3_ as a typical procedure following Equation (2) [40]. The XRD patterns and SEM images of the as-synthesized LaB_6_ nanopowders are shown in Figure 2. The XRD patterns (Figure 2a) confirm that only the LaB_6_ phase (PDF#97-060-2780) is detected in the powders synthesized at different temperatures, confirming a complete reaction. The full width at half maximum (FWHM) of the (110) peaks decreased as the synthesis temperature increased from 800 °C to 1300 °C, indicating a gradual growth in particle size. The SEM images (Figure 2b–g) show the evolution of LaB_6_ particle morphology and particle size with increasing temperature. As the synthesizing temperature increased from 800 °C to 900 °C, the as-produced LaB_6_ powder was a typical agglomeration formed by irregularly shaped particles, with an average particle size of less than ~50 nm. At 1000 °C, cubic particles began to emerge, and a well-crystallized cubic morphology dominated when the synthesizing temperature reached 1300 °C. In addition, the average particle size of the LaB_6_ powders synthesized at 1300 °C reached ~300 nm, indicating abnormal particle growth accompanied by a broad size distribution.

Similar to Equation (1), the complete reaction between ZrO_2_ and NaBH_4_ is displayed in Equation (4) [44]:

(4) $$ZrO_{2}\;+\;3NaBH_{4}\to ZrB_{2}\;+\;NaBO_{2}\;+\;2Na\;+\;6H_{2}$$

**Figure 3:**
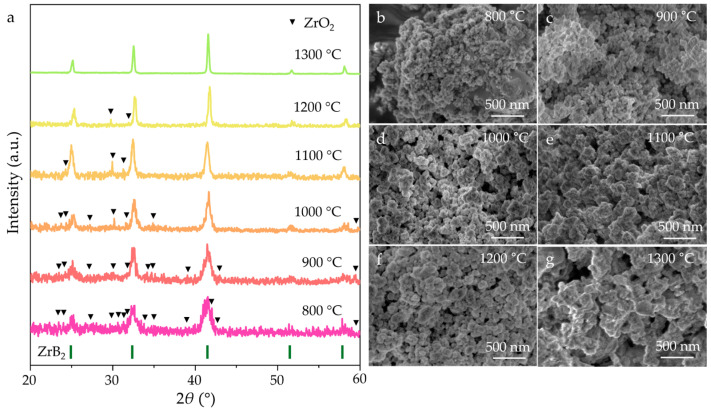
(**a**) XRD patterns and (**b**–**g**) SEM images of ZrB_2_ nanopowders synthesized at 800–1300 °C.

For the ZrB_2_ (PDF#97-061-5772) nanopowders synthesized at 800–1200 °C, the diffraction peaks of ZrO_2_ still existed (Figure 3a), which is due to nanoparticle agglomeration and the high melting point of ZrO_2_. The complete reaction between ZrO_2_ and NaBH_4_ occurred when the synthesizing temperature reached 1300 °C. The FWHM of the ZrB_2_ peaks gradually decrease with increasing synthesizing temperature, implying slow particle growth during synthesis. The SEM images (Figure 3b–g) revealed that all the particles in the ZrB_2_ powders synthesized at different temperatures exhibited an irregular morphology. Notably, the particle size remained at a relatively low value within the nanometer range (<70 nm) despite elevated sintering temperatures. These results demonstrate that the synthesizing temperature significantly influences the phase purity and morphological features of the final products.

**Figure 4:**
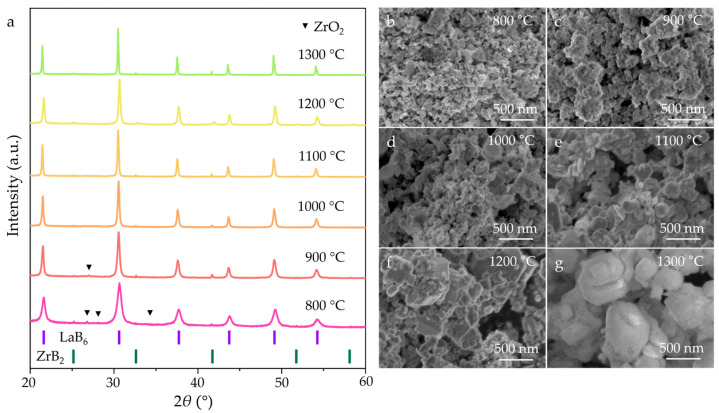
(**a**) XRD patterns and (**b**–**g**) SEM images of LaB_6_-ZrB_2_ nanopowders synthesized at 800–1300 °C.

Based on the synthesis of single-phase LaB_6_ and ZrB_2_, LaB_6_-ZrB_2_ composite powders were successfully prepared via an in situ simultaneous reduction of La_2_O_3_, ZrO_2_, and NaBH_4_, with a weight ratio of LaB_6_:ZrB_2_ = 79:21. As shown in Figure 4a, the XRD analysis reveals that the LaB_6_, ZrB_2_, and residual ZrO_2_ peaks are detected at 800–900 °C. However, beyond 900 °C, the residual ZrO_2_ peaks become undetectable. This result indicates that the initial temperature for the complete reaction between ZrO_2_ and NaBH_4_ in the synthesis of LaB_6_-ZrB_2_ nanopowders is lower than that in synthesis of ZrB_2_ nanopowders. This reduction is attributed to the eutectic interaction between LaB_6_ and ZrB_2_, which enhances the reaction kinetics by lowering the activation energy. The in situ reaction can be summarized as follows:

(5) $${La}_{2}O_{3}\;+\;ZrO_{2}\;+\;16NaBH_{4}\to 2LaB_{6}\;+\;ZrB_{2}\;+\;NaBO_{3}\;+\;NaBO_{2}\;+\;14Na\;+\;32H_{2}$$

**Figure 5:**
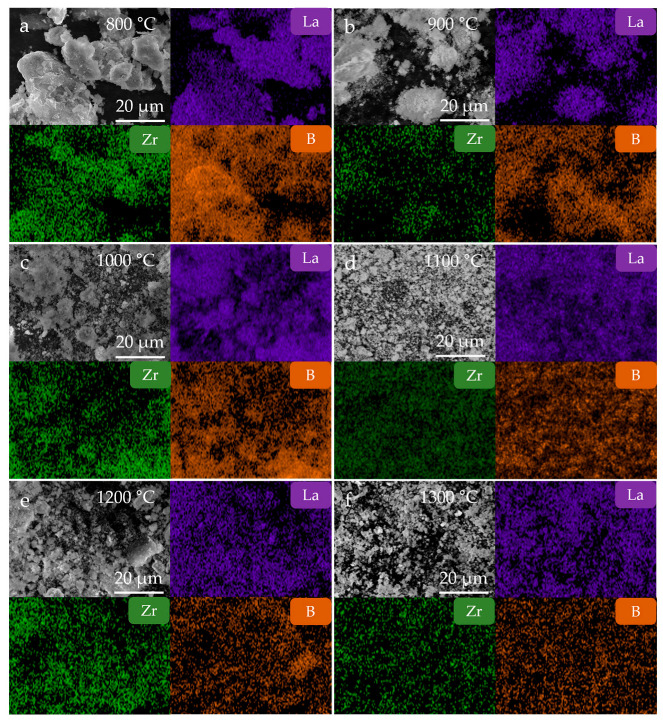
The SEM images and the corresponding EDS mappings of LaB_6_-ZrB_2_ nanopowders synthesized at (**a**) 800 °C, (**b**) 900 °C, (**c**) 1000 °C, (**d**) 1100 °C, (**e**) 1200 °C and (**f**) 1300 °C.

The SEM images (Figure 4b–g) of the LaB_6_-ZrB_2_ composite powders at various temperatures reveals particle growth behavior. At 800–1000 °C, the particles exhibited a polyhedral morphology, and their size gradually increased with rising temperature. At 1100 °C, cubic LaB_6_ particles form, and ZrB_2_ particles are distributed homogeneously around them. It should be noted that this attachment phenomenon is particularly evident when the synthesis temperature reaches 1200 °C, demonstrating uniform phase distribution. As displayed in EDS mappings of the LaB_6_-ZrB_2_ nanopowders synthesized at different temperatures (Figure 5), rare earth element La, transition metal element Zr, and element B are uniformly distributed in the composite boride nanopowders without obvious segregation, further confirming the advantages of the LaB_6_-ZrB_2_ composite nanopowders prepared using this method.

**Figure 6:**
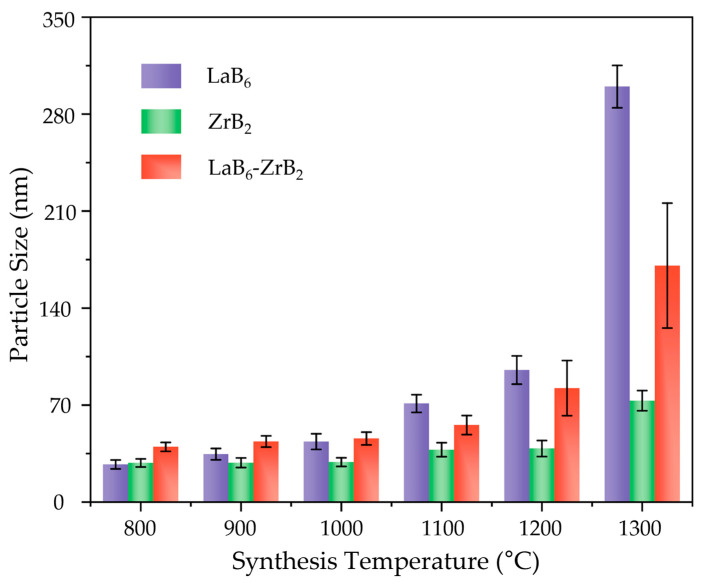
The average particle sizes of LaB_6_, ZrB_2_ and LaB_6_-ZrB_2_ nanopowders synthesized from 800–1300 °C.

To comprehensively evaluate the effect of temperature on the particle sizes of single-phase and composite nanopowders, the average particle sizes of LaB_6_, ZrB_2_, and LaB_6_-ZrB_2_ synthesized at 800–1300 °C are summarized in Figure 6. The average particle sizes were obtained by randomly selecting 4–6 SEM images of each powder and measuring 100–200 particles in different areas of each image. The average particle sizes of these three nanopowders increased gradually with the increase in synthesizing temperatures. Below 1100 °C, the average particle size of the composite powders is higher than that of the single-phase powders, suggesting that the eutectic interaction facilitates the synthesis reaction while providing additional energy for grain growth. As the temperature reaches 1100 °C, the average particle size of the composite nanopowders is between the LaB_6_ and ZrB_2_ single phases due to abnormal particle growth of LaB_6_.

**Figure 7:**
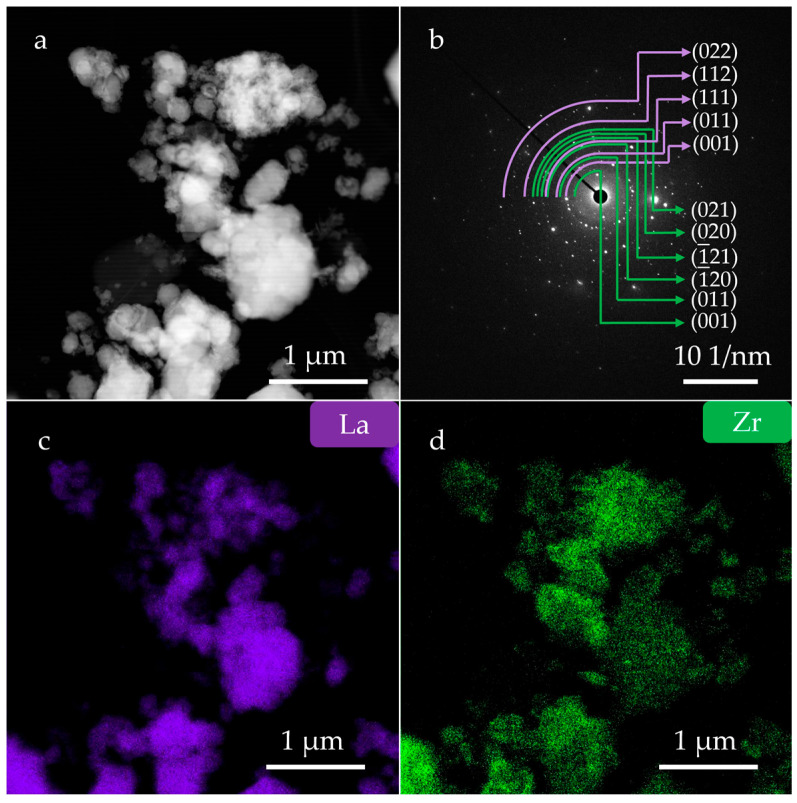
(**a**) TEM image of LaB_6_-ZrB_2_ nanopowders, (**b**) the corresponding SAED pattern of (**a**), and (**c**,**d**) the corresponding EDS maps of (**a**).

**Figure 8:**
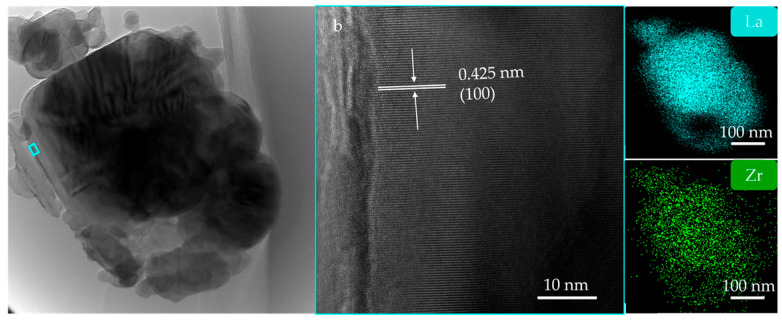
(**a**) HRTEM image of LaB_6_-ZrB_2_ nanopowders, (**b**) partial enlargement of the box in (**a**), and (**c**,**d**) corresponding EDS compositional maps of (**a**).

TEM analysis was performed to determine the microstructure and compositional uniformity of the LaB_6_-ZrB_2_ nanopowder obtained at 1100 °C. The dark field TEM micrograph was displayed in Figure 7a. Slightly agglomerated and nanoscaled particles were observed. As shown in Figure 7b, the corresponding SAED patterns confirm the coexistence of LaB_6_ and ZrB_2_, where the purple arcs indicate the diffraction rings of LaB_6_ and the green indicate ZrB_2_. The EDS mappings are shown in Figure 7c,d and demonstrate the uniform phase distribution of LaB_6_ and ZrB_2_. Figure 8a displays a high-resolution transmission electron microscopy (HRTEM) image. It can be observed that the cubic particles are tightly attached to irregular particles, resulting in a small aggregate. Figure 8b shows a partially enlarged image of the blue box in Figure 8a. It displays a periodic lattice structure with *d*-spacing of 0.425 nm, corresponding to the (100) plane of LaB_6_ (0.415 nm, PDF#97-060-2780). Figure 8c,d show the EDS mappings of the LaB_6_-ZrB_2_ nanopowders in Figure 8a, which further demonstrate the uniform phase distribution.

**Figure 9:**
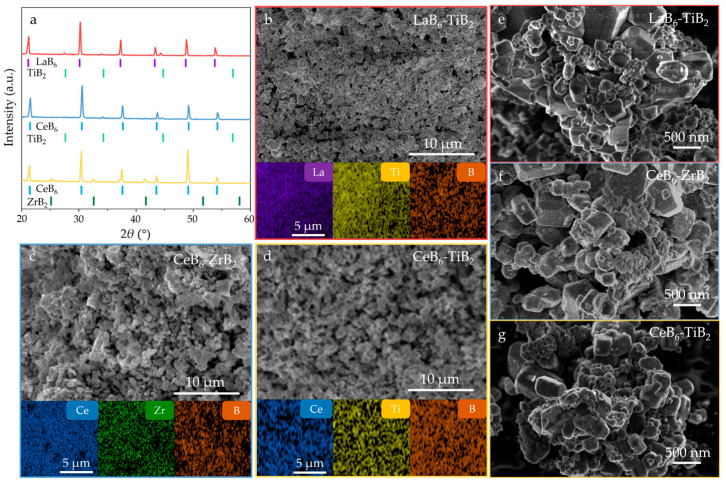
(**a**) XRD patterns, (**b**–**d**) SEM images and EDS mappings, and (**e**–**g**) the high magnifications of LaB_6_-TiB_2_, CeB_6_-ZrB_2_, and CeB_6_-TiB_2_ composite nanopowders obtained in situ at 1300 °C.

**Figure 10:**
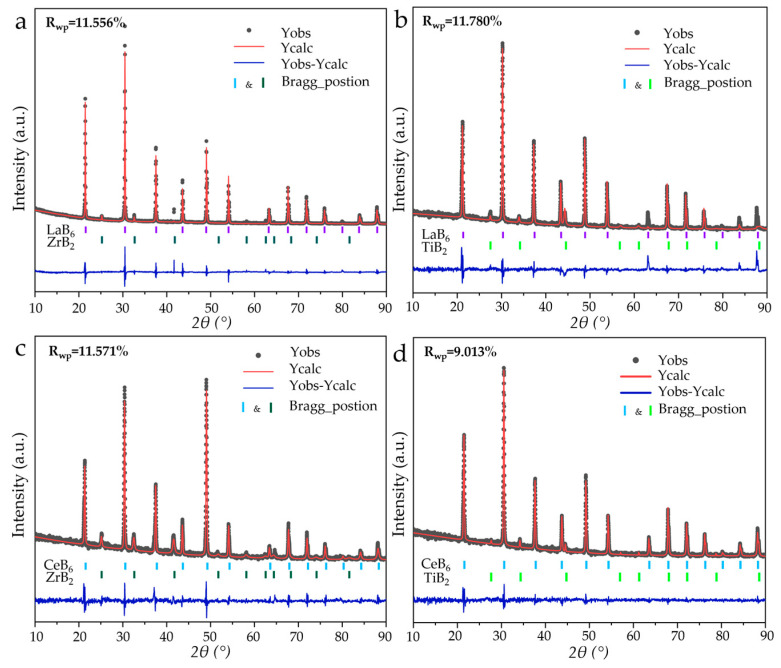
Rietveld refinement of synthesized composite nanopowders: (**a**) LaB_6_-ZrB_2_; (**b**) LaB_6_-TiB_2_; (**c**) CeB_6_-ZrB_2_; and (**d**) CeB_6_-TiB_2_.

**Figure 11:**
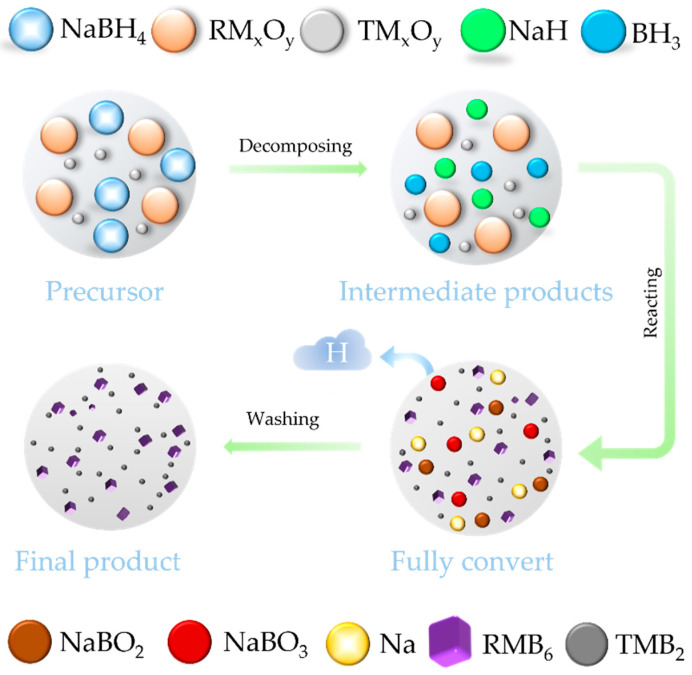
Reaction mechanism schematic of the metal oxides and NaBH_4_.

According to Equation (5), the LaB_6_-TiB_2_ (TiB_2_: PDF#97-003-0330), CeB_6_-ZrB_2_ (CeB_6_: PDF#97-019-4318), and CeB_6_-TiB_2_ composite nanopowders were successfully synthesized with a eutectic weight ratio (RMB_6_:TMB_2_ = 79:21). As exhibited in Figure 9a, XRD analysis confirms that all composite nanopowders synthesized at 1300 °C are composed of the target product without any impurities and by-products, indicating the feasibility of this synthesis pathway. The SEM and EDS mappings (Figure 9b–g) reveal uniform nanostructured morphologies without elemental agglomeration or segregation, which also demonstrates the effectiveness of the in situ method in achieving a homogeneous phase distribution. Figure 9e–g displays the high-magnification SEM images of the LaB_6_-TiB_2_, CeB_6_-ZrB_2_, and CeB_6_-TiB_2_ composite nanopowders, and it can be observed that these three products possess nanosized particles. The refinement results of the XRD patterns of the LaB_6_-ZrB_2_, LaB_6_-TiB_2_, CeB_6_-ZrB_2_, and CeB_6_-TiB_2_ nanopowders are shown in Figure 10. The R_wp_ values of the diffraction patterns obtained via calculations are 11.556, 11.780, 11.571, and 9.013, respectively, indicating the reliability of the refinement results. In addition, lattice constants and phase weight ratios were also calculated. The lattice constant of LaB_6_ is 0.420 nm in LaB_6_-ZrB_2_ and LaB_6_-TiB_2_ powders, which is close to the theoretical counterpart. The measured *d*-spacing of the (100) plane, as displayed in Figure 8, is larger than the theoretical value of 0.415 nm, which might be caused by the lattice distortion. The calculated weight ratios are LaB_6_:ZrB_2_ = 89.2:10.8, LaB_6_:TiB_2_ = 82.3:17.7, CeB_6_:ZrB_2_ = 81.8:18.2, and CeB_6_:TiB_2_ = 81.2:18.8, which are in agreement with the eutectic weight ratio of 79:21, further confirming the feasibility of this method. As depicted in Figure S1, the Raman spectra only detected the existence of LaB_6_, CeB_6_, and TiB_2_ without any other phase, and ZrB_2_ has no peaks because it is not Raman active. Based on the current study, the in situ formation mechanism of RMB_6_-TMB_2_ composite powders is summarized and presented in Figure 11. It is obvious that the decomposition of NaBH_4_ plays a critical role in this process by simultaneously providing both a boron source and reducing agents. The release of BH_3_ during NaBH_4_ decomposition enhances its accessibility to metal oxide surfaces, facilitating uniform boride nucleation. Therefore, it ensures that the synthesized composite powders remain at the nanoscale level while achieving uniform distribution of RMB_6_ and TMB_2_.

## 4. Conclusions

In this work, RMB_6_-TMB_2_ composite nanopowders were synthesized in situ via a one-step solid-state reduction method using metal oxides and NaBH_4_ as raw materials. The LaB_6_-ZrB_2_ composite nanopowders with a eutectic weight ratio exhibited accelerated reaction kinetics compared to their single-phase counterparts. The EDS mappings of the HRTEM images demonstrated that the phase distribution of the composite powders is uniform even at the nanoscale level. The refinement results prove that the lattice constant of the composite powder is close to the theoretical value, and the obtained mass ratio is in agreement with the designed eutectic ratio. This method eliminates the phase separation and contamination that exists with conventional manual mixing, which provides the feasibility for designing advanced boride composites of RMB_6_-TMB_2_ with excellent thermionic and mechanical properties.

## Supplementary Materials

The following supporting information can be downloaded at https://www.mdpi.com/article/10.3390/nano15171341/s1: Figure S1: Raman spectras of (a) LaB_6_-ZrB_2_, (b)LaB_6_-TiB_2_, (c) CeB_6_-ZrB_2_ and (d) CeB_6_-TiB_2_. (▲: LaB_6_, ■: CeB_6_ and ●: TiB_2_).
