# Supplementary material for: In Situ Synthesis of RMB6-TMB2 Composite Nanopowders via One-Step Solid-State Reduction
Source: Nanomaterials (Basel). 2025 Sep 1;15(17):1341. doi: 10.3390/nano15171341 (PMC12430549; doi:10.3390/nano15171341)
Supplement: Supplementary file 1 [file nanomaterials-15-01341-s001.zip › nanomaterials-3750638-supplementary.pdf]

nanomaterials

Supplementary Information

In Situ Synthesis of RMB_6-TMB_2 Composite Nanopowders via One-Step Solid-State Reduction

Xiaogang Guo [1], Linyan Wang [2], Hang Zhou [1], Jun Xu [1], An Liu [3], Mengdong Ma [3], Rongxin Sun [4], Weidong Qin [1], Yufei Gao [1], Bing Liu [1,*], Baozhong Li [1], Lei Sun [1,*] and Dongli Yu [1]

[1] Center for High Pressure Science (CHiPS), State Key Laboratory of Metastable Materials Science and Technology, Yanshan University, Qinhuangdao 066004, China; guoxiaogang1994@stumail.ysu.edu.cn (X.G.); zh18993920050@stumail.ysu.edu.cn (H.Z.); xujun0924@stumail.ysu.edu.cn (J.X.); weidong07080814@stumail.edu.cn (W.Q.); gyf@ysu.edu.cn (Y.G.); lbz@ysu.edu.cn (B.L.); ydl@ysu.edu.cn (D.Y.)

[2] State Key Laboratory of Advanced Space Propulsion, Space Engineering University, Beijing 101416, China; wanglinyan@hgd.edu.cn

[3] State Key Laboratory of Crane Technology, Yanshan University, Qinhuangdao 066004, China; liuan@stumail.ysu.edu.cn (A.L.); mamengdong@ysu.edu.cn (M.M.)

[4] Super Hard Material Industry Technology Research Institute, Zhengzhou 450000, China; sunrongxin@hnas.ac.cn

* Correspondence: 9820230042@nankai.edu.cn (B.L.); leisun@ysu.edu.cn (L.S.)

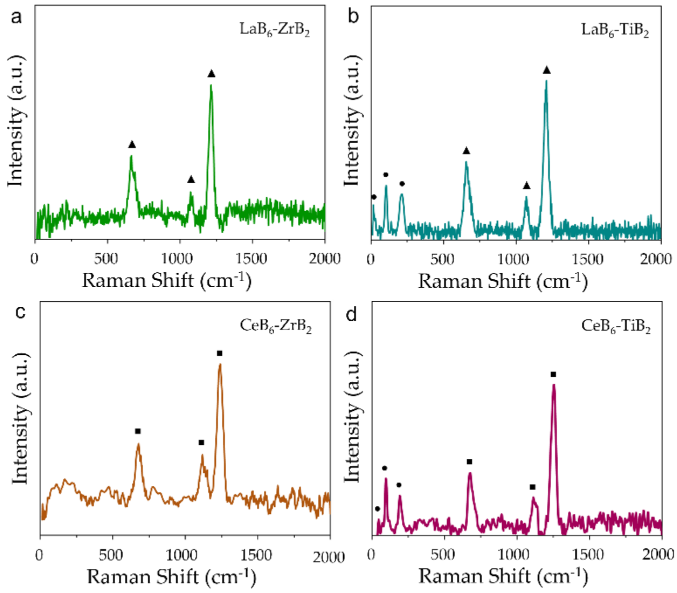

Figure S1. Raman spectras of (a) LaB_6-ZrB_2, (b)LaB_6-TiB_2, (c) CeB_6-ZrB_2 and (d) CeB_6-TiB_2. (▲: LaB_6, ■: CeB_6 and ●: TiB_2).
